# Evaluation of the Management of Febrile Neutropenia in a Tertiary Care Center

**DOI:** 10.1155/cjid/3681955

**Published:** 2025-04-08

**Authors:** Farah Feghali, Carl Aoun, Wissam K. Kabbara

**Affiliations:** Department of Pharmacy Practice, School of Pharmacy, Lebanese American University (LAU), Byblos, Lebanon

## Abstract

**Introduction:** Febrile neutropenia (FN) is defined by an absolute neutrophil count (ANC) less than or equal to 500 cells per microliter with a concurrent single oral body temperature of more than or equal to 38.3° C (orally) or more than 38.0° C maintained over one hour. There are different published guidelines for the management of FN. The primary objective of this study is to assess the management of FN in terms of choice, dose, and duration of empirical antimicrobial therapy and postculture results in our tertiary medical center.

**Methods:** This is a retrospective review of medical records for patients admitted to the institution with a diagnosis of FN between 2018 and 2023. An electronic review of the medical records of all adult patients admitted with the diagnosis of FN was conducted through the platform of the medical records. A set of criteria for the evaluation of therapy was developed based on standard local and international guidelines.

**Results:** The study included 280 patients who fit the inclusion/exclusion criteria. Around half of the patients did not have a focus of infection (48.9%). The overall treatment regimen was appropriate in only 32.1% of the patient cases which includes an appropriate choice, dose, and duration of therapy. The choice of the antimicrobial(s) was inappropriate in 49.3% of the patient cases. The dosing regimen and treatment duration were inappropriate in 36.5% and 19.3% of the patient cases, respectively.

**Conclusion:** This study evaluated the appropriate management of FN at our medical center including the appropriate choice, dose, and duration of the antimicrobials used. The overall management of FN was inappropriate in two thirds of the patient cases (67.9%) when evaluated according to the assessment criteria. The choice, dose, and duration of treatment needs improvement for optimization of therapy and improvement of patient outcomes.

## 1. Introduction

Cancer patients receive intensive chemotherapy treatment regimens, predisposing them to a decrease in their white blood cell production from the bone marrow. It also disrupts their mucosal flora, exposing them to a higher risk of acquiring infections [1]. Fever is usually the first indicative sign of infection in this patient population [2]. The National Comprehensive Cancer Network (NCCN) defines febrile neutropenia (FN) as an absolute neutrophil count (ANC) less than or equal to 500 per μL (or less than or equal to 1000 per μL with a predicted decline to less than or equal to 500 per μL over the next 48 h) with a concurrent single oral body temperature of more than or equal to 38.3° (orally) or more than 38.0 degrees Celsius maintained over one hour [3]. This definition is compliant with the FN definition of the Infectious Disease Society of America (IDSA) [4].

There are multiple factors included in the assessment and treatment of patients with FN to decide on the antimicrobial regimen they should be prescribed and the appropriate duration of their treatment [5]. Studies indicate that the response to an antimicrobial treatment is directly related to the immunologic state of the patient and the ANC level. The lower the ANC is, the worse the patient's prognosis is in terms of infection resolution. Moreover, some patients can be eligible for outpatient antimicrobial therapy, depending on their ANC, the severity of their symptoms as well as their comorbidities. The Multinational Association for Supportive Care in Cancer (MASCC) helps stratify outpatients with FN [6]. FN patients with low MASCC scores usually have worse clinical outcomes and should be empirically treated with intravenous (IV) antibiotics and, therefore, an inpatient treatment regimen [7]. FN patients with a MASCC score higher than 21 can be managed safely and effectively in an outpatient setting with oral antibiotics such as amoxicillin/clavulanic acid and ciprofloxacin.

After assessing the patient, the first-line management of FN would include broad-spectrum empirical antibiotic(s) until the source of the infection is determined [5]. In Lebanon, the most isolated microorganisms include coagulase-negative *Staphylococci*, *Escherichia coli*, and *Klebsiella* species [8]. There is also an increased prevalence of extended spectrum beta-lactamases (ESBL)-producing organisms following the most recent antibiogram of the Lebanese American University Medical Center–Rizk Hospital (LAUMC-RH), which should be taken into consideration. According to the NCCN and IDSA guidelines on the management of FN, patients presenting with the criteria of FN should be managed with IV broad spectrum antibiotics such as cefepime, piperacillin–tazobactam, or an antipseudomonal carbapenem, sometimes requiring a combination of more than one antibiotic. The combination of antibiotics would include an antipseudomonal agent with a fluoroquinolone (ciprofloxacin or levofloxacin) or an aminoglycoside [4].

According to the Lebanese Society of Infectious Diseases and Clinical Microbiology Guidelines on the Management of FN (LSIDCM), the treatment options for the management of FN include either a carbapenem monotherapy as a first-line agent or the combination of an antipseudomonal agent with either an aminoglycoside or a fluoroquinolone, as broad spectrum first-line agents. When indicated, the guidelines require the addition of vancomycin or teicoplanin to cover methicillin-resistant *Staphylococcus aureus* (MRSA). Vancomycin or teicoplanin can also be added after 24–48 h of initiating antibiotics and worsening patient clinical status [4]. For potential *Clostridiodes difficile* infections, oral vancomycin or fidaxomicin should be added. Other factors that might affect the choice of antimicrobials would be the presence of necrotizing ulcers or vesicular lesions where acyclovir would be added to the treatment for suspected herpes zoster virus (HZV) or varicella zoster virus (VZV) infections. The criteria for the initiation of colony growth stimulating factors (G-CSFs) are commonly unclear in all the guidelines and depend on patient specific risk factors such as previous episodes of FN, an ANC less than 100, a documented infection, or an age above 65 years. Since FN comes with a high risk for mortality and morbidity depending on the patient's condition, it is important to evaluate and study the pattern of treatment of FN in our tertiary care center at LAUMC-RH. This would help identify gaps for potential areas of improvement of patient care, resulting in better clinical outcomes of FN among our cancer patients. There are differences in the choice of antimicrobials and the duration and doses of the regimens with a need to balance between the treatment efficacy, safety, and antimicrobial resistance.

This study evaluated our institution's current treatment approaches to develop a practice protocol for the management of FN at the institution in order to reduce the consumption of broad-spectrum antibiotics and improve patient outcomes. The primary objective of this study was to evaluate the appropriateness of FN management at our institution. The compliance to the guidelines for the management of FN at our institution was assessed after reviewing the guidelines of the following societies: IDSA, LSIDCM, and NCCN. The regimens were evaluated pre- and postculture results for potential de-escalation or required upescalation of antimicrobial therapy.

## 2. Materials and Methods

### 2.1. Setting and Design

This was a retrospective review of medical records for patients admitted to the institution with a diagnosis of FN between 2018 and 2023. The medical records of 350 patients admitted to LAUMC-RH with a diagnosis of FN were evaluated. The sample size included 280 (*n* = 280) admitted patients to LAUMC-RH with a diagnosis of FN. This descriptive observational study was conducted in a tertiary 194-bed teaching hospital over a 5 year period. Data of the patients admitted for the management of FN to the institution were collected using a data collection form that included demographic patient data, clinical measurements, type of cancer, chemotherapy regimen, antimicrobial agents received with the dose and the duration of the treatment, cultures taken and sensitivities, any change in antimicrobial regimen, and use of G-CSF.

### 2.2. Inclusion/Exclusion Criteria

Of the 350 patients with FN, 280 satisfied the age and FN definition inclusion criteria. Subjects eligible for enrollment included males and females aged > 18 years with a diagnosis of FN. FN was defined in a patient with ANC less than or equal to 500 (or less than or equal to 1000 with a predicted decline to less than or equal to 500 over the next 48 h) with concurrently having either a single oral body temperature of more than or equal to 38.3 degrees Celsius (orally) or more than 38.0° maintained over one hour.

Patients were classified according to standardized criteria into 3 categories: microbiologically defined infection, which included patients with bacteremia or nonbacteraemic patients with a defined focus of infection who have an associated pathogen, clinically defined infection, which included patients with a focus of infection but without microbiologic pathogenesis (either could not be proven or was inaccessible to examination), and unexplained fever, which included patients who developed a new fever that was not accompanied by either clinical or microbiologic evidence of infection. Clinical evidence of infection would include fever, constitutional symptoms, or satisfaction of the criteria for systemic inflammatory response syndrome (SIRS) even without a definable clinical focus.

An electronic review of the medical records of all the patients admitted with the diagnosis of FN was conducted through the platform of the medical records (LASERFICHE) at the institution. The study was approved by the Institutional Review Board (IRB) at LAU and was conducted in accordance with the Declaration of Helsinki. The assessment of the treatment of FN was evaluated both empirically and after the results of the cultures were available, if positive. The analysis included all the data collected from the charts of the patients since their first dose of antimicrobials until the discontinuation of all agents, or their discharge from the hospital. Informed consent was not required since it was a retrospective study. There are no subject identifiers used in the final manuscript or data collection form to respect the confidentiality of the participants.

### 2.3. Assessment Criteria

Assessment criteria were developed based on the IDSA, LSIDCM, and NCCN guidelines for the management of FN, tailored to the epidemiology of the institution and based on the clinical judgment for each patient case (Appendix). First-line treatment included a broad-spectrum antibiotic such as cefepime, meropenem, imipenem–cilastatin, or piperacillin–tazobactam. In patients with severe beta-lactam allergy, initiation of vancomycin plus aztreonam was recommended. In patient cases where a Gram-positive coverage was needed (history of MRSA, suspected catheter or skin and soft tissue infection, sepsis/hypotension, previous hospitalization or receipt of IV antibiotics during the past 90 days) or if the patient remained unstable and febrile for the next 24–48 h, vancomycin or teicoplanin was added. If the patient remained unstable for 72 h, an antifungal was added appropriately. Double antipseudomonal coverage (addition of an aminoglycoside or ciprofloxacin/levofloxacin) was recommended for patients who were hospitalized during the past 90 days or who received a course of IV antibiotics during the past 90 days.

The dose adjustment was conducted based on the protocol of renal adjustment for antimicrobials used at the institution. The protocol was developed based on the package insert and relevant published guidelines. In cases of skin and soft tissue infection, patients were treated for 5–14 days and in cases of bloodstream infections, patients were treated for an average of 7–14 days. For bacterial pneumonia, patients were treated for 5–14 days and for candidemia patients were treated for 2 weeks after the first negative culture.

When no focus of infection was identified, patients were treated until clinically stable and with an ANC higher than 500 and expected to further increase. After assessing the agents used for the management of FN in the different patients, the escalation or de-escalation of antimicrobials was evaluated based on the results of the cultures and hemodynamic stability of the patients (improvement in the stability of the blood pressure, temperature, and clinical improvement). The overall compliance to the guidelines treatment regimen included an appropriate choice, appropriate dose, and appropriate duration of the antimicrobial (s). The appropriateness of each component was also evaluated individually. The use of G-CSF throughout the patients' treatments was reported as well in the results.

### 2.4. Statistical Analysis

The data were processed using the Statistical Package for Social Sciences (SPSS, Version 28) software. The responses were tabulated and cross-tabulated, and the percentages, median, and averages were calculated.

## 3. Results

### 3.1. Baseline Characteristics

The study included 280 patients who fit the inclusion/exclusion criteria. About half of the patients had unexplained fever (48.9%). In patients with microbiologically defined infections, the most common foci were upper and lower respiratory tract infections (18.2%) followed by intra-abdominal infections (11.4%). Other less common infections included skin and soft tissue infections (5.0%) and urinary tract infections (3.9%).

A summary of the demographic information of the patients is represented in Table 1. Most of the patients admitted for FN were male (56.7%). The median age of the patients was 66 years old and ranged between 19 and 96 years of age. Most of the patients presented with an ANC between 100 and 500 (38.2%). The majority had a hematologic malignancy (68.9%).

### 3.2. Appropriateness of the Empirical Treatment

The overall treatment regimen was appropriate in 32.1% of the patient cases, which includes an appropriate choice, appropriate dose, and appropriate duration of the antimicrobial(s) (Table 2). Evaluating each component individually, the choice of antimicrobial was appropriate in 50.7% of the patient cases, the dose was appropriate in 63.5% of the patient cases, and the duration of therapy was appropriate in 80.7% of the patient cases.

For individual components of the primary endpoint, the choice of the antimicrobial(s) was inappropriate in 138 of the patient cases. Patients received an inappropriate addition of Gram-positive coverage, antifungal agent, or antiviral agent and sometimes a broader spectrum of antibiotics than required. More than 80% of the patients received meropenem as part of their broad-spectrum empiric antibiotic regimen for the management of FN. The second and third most prescribed antimicrobials were teicoplanin (32.1%) and vancomycin (22.1%).

The dosing regimen was inappropriate in 102 patient cases with 49 patient cases pertinent to teicoplanin (Table 3). Teicoplanin was not dosed properly based on the patient's weight. Vancomycin dosing was inappropriate in terms of weight-based dosing in 9.6% of the patient cases. The remaining antimicrobials were not dosed properly according to the patients' kidney function. Teicoplanin trough concentrations were not monitored on any patient since the monitoring kits are not available in Lebanon. All patients on vancomycin were monitored according to the institution's protocol.

In 54 patient cases, the duration of the treatment was inappropriate with only six patient cases receiving a treatment shorter than recommended and 48 patients receiving a longer duration of treatment. In most patient cases, bloodstream infections were treated for more than 7–14 days (21 out of 48 patient cases). Also, 11 out of 48 patients were treated for UTIs for longer than the optimal duration of treatment. Finally, 13 out of 48 patients completed a longer course of antibiotics with no focus of infection, acceptable ANC level, and good clinical condition.

The individual appropriateness of the choice, dose, and duration of teicoplanin and vancomycin are represented in Table 4. A total of 90 and 62 patients out of 280 received teicoplanin and vancomycin, respectively.

### 3.3. Appropriateness of Therapy Postculture Results

Most of the patients presenting with FN did not have a clear focus of infection. A total of 159 positive cultures were reported in 103 patients. The most isolated microorganisms were *Escherichia coli* (33.3% of the cultures) and *Klebsiella pneumoniae* (11.3% of the cultures). A total of 22.6% of the *Escherichia coli* strains and 11.1% of the *Klebsiella pneumoniae* strains were ESBL-producing. A total of 11.1% of the *Klebsiella pneumoniae* strains were carbapenem resistant. *Pseudomonas aeruginosa* was reported in 9.0% of the cultures. Bloodstream infections were the most reported source of infection for patients with positive cultures. *Staphylococcus aureus* was isolated in 5 out of the 159 cultures; 40% of which were methicillin-resistant strains.  Table 5 indicates the appropriateness of treatment by site of infection and causative microorganism.

As for the treatment modification, after the cultures came back positive in 103 patients, there had been few modifications to the antimicrobials to provide a tailored regimen based on the sensitivities of the microorganisms. In 33.0% (34/103) of these patient cases, the treatment was de-escalated to an appropriate narrower antimicrobial based on the sensitivity of the microorganism. In 9.7% (10/103) of these patient cases, a more appropriate escalation to a broader spectrum antimicrobial was performed to cover the cultured microorganism. In 45.6% (47/103) of the patient cases with positive cultures, a de-escalation to a narrow and more specific antibiotic was more appropriate but not completed. In 11.7% (12/103) of the patient cases with positive cultures, an escalation to a broader antibiotic was needed but not completed.

### 3.4. Length of Hospital Stay/Defined Daily Doses/Side Effects

The length of hospital stay was significantly longer for patients who were noncompliant (11.5 ± 4.2 days) to guidelines as compared with patients who were compliant to guidelines (8.7 ± 2.7 days) (*p*=0.032). Patients who received inappropriate therapy had a significant higher utilization of antibiotics (*p* < 0.001). Total use of antibiotics expressed as number of defined daily doses (DDDs) per 1000 patients per day was 32 for patients who received appropriate therapy versus 47 for patients who received inappropriate therapy. The incidence of *Clostridiodes difficile* was significantly higher in the inappropriate treatment group (16 patients) as compared with the appropriate treatment group (7 patients) (*p*=0.022). The incidence of antibiotic-induced renal impairment and intensive care unit admission was comparable between the two groups. The study was not powered to detect significant differences in mortality/survival rate as only few patients have died during hospital stay (6 in the inappropriate treatment group vs. 4 in the appropriate treatment group).

## 4. Discussion

This is the first study conducted at LAUMC-RH to evaluate the appropriateness of the management of FN in terms of choice, dose, and duration of therapy. It includes an assessment on the appropriate modification of therapy based on the results of the cultures (when positive). Based on the results of the study, 32.1% of the patient cases received an overall appropriate regimen. The empirical antimicrobial choice was appropriate in 50.7% of the patient cases, the dose was appropriate in 63.5% of the patient cases, and the duration of therapy was appropriate in 80.7% of the patient cases. A similar study that evaluated the empirical treatment of FN at UC Health Memorial Hospital, showed that the institution was not fully compliant with their guidelines for the management of FN; only 43.8% of their patient cases received an appropriate treatment regimen [9]. In terms of the types of malignancy, Cherri et al. described leukemia as the most reported malignancy among cancer patients in Lebanon (29.7%), reflected also in this study with 68.9% of the patients presenting with hematologic malignancy [10].

The inappropriate choice of antimicrobials was related to the addition of MRSA coverage when not necessary or an inappropriate addition of antifungal or antiviral to the empiric treatment of the patients when not indicated. Another study conducted in Lebanon for the assessment of FN management concluded that 86.2% of the patient cases enrolled received an appropriate treatment regimen [10]. In the same study, there was inappropriate addition of an antifungal and/or antiviral agent. The most commonly prescribed antibiotic in our study was meropenem followed by teicoplanin and vancomycin. The most prescribed empirical antibiotic was cefepime or cefepime plus vancomycin followed by meropenem [10].

The dosing of antimicrobials was inappropriate in 36.4% of patient cases. Vancomycin weight-based dosing was inappropriate in 9.7% of the patient cases, while teicoplanin dosing was inappropriate in 54.4% of the patient cases because the dosing approach did not rely on the actual body weight of the patient. The remainder of the inappropriate dosing required renal dose adjustment following the protocol of the hospital for dose adjustment of antimicrobials. The trough levels of vancomycin were monitored in all cases; however, as there are no teicoplanin monitoring kits available in Lebanon; therefore, therapeutic drug monitoring for teicoplanin was not performed and the dose was evaluated depending on the actual body weight and renal function of the patient. The inappropriate dosing related to teicoplanin was mainly due to using a standard dose for all patients rather than the more accurate weight-based dosing.

According to Keck et al., the guidelines recommend discontinuing the antimicrobial treatment when the ANC is above 500 cells per microL while taking into consideration the clinical stability of the patient and time to defervescence [11]. The duration of the treatment was inappropriate in 19.3% of the patient cases. This exposed the patients to a longer duration of antibiotics than needed leading to the potential risk of side effects and emergence of resistance. In this study, patients who did not have a positive culture with a clear focus of infection were treated until the resolution of clinical signs and symptoms of FN, or the resolution of neutropenia.

The choice of vancomycin was appropriate in 85.5% of our patient cases. According to Goldsmith et al., only 25% of the patient cases received an appropriate addition of vancomycin [9]. The addition of vancomycin is appropriate in the cases of catheter-related infections, soft tissue infections, or those with known colonization with drug-resistant Gram-positive microorganisms, and hemodynamic instability [11].

In our study, 103/280 (36.8%) patients had at least one positive culture. Of the positive cultures, 32.7% (52 out of 159 positive cultures) were blood cultures. A similar study conducted in Lebanon on patient cases admitted for FN due to bloodstream infections showed 9.3% were caused by Gram-negative multidrug resistant organisms [12]. Cherri et al. also reported blood infections as the most common focus of infection in FN [10]. Another study conducted by Kanafani et al. showed that gastrointestinal infections were predominant (31.6% of patient cases) and that 23.7% of the patient cases had negative cultures [13]. In this study, 63.2% of the patient cases had no focus of infection. as compared to 48.9% of the patient cases in our study.

The length of hospital stay was longer in patients who were noncompliant to guidelines; and these patients utilized more antibiotics per the defined daily doses per 1000 patient days. Noncompliance with published guidelines for antimicrobial therapy can significantly impact patient outcomes in various ways. First, inappropriate use of antibiotics can lead to treatment failure, prolonging the patient's illness or exacerbating their condition [14]. This may result in unnecessary hospitalizations, increased healthcare costs, and increased mortality. Furthermore, noncompliance to guidelines can contribute to the development of multidrug-resistant microorganisms, a critical global health concern that increases patient morbidity and mortality [15]. Adherence to published guidelines ensures that the appropriate antibiotics are used at the correct doses and for the recommended duration; thereby optimizing therapeutic effectiveness and minimizing adverse effects [16]. In addition, failure to follow these guidelines can strain healthcare resources, as resistant infections require more intensive treatment strategies, often leading to longer hospital stays and more complex care.

There were several limitations to this study. First, it was a retrospective review of patient medical charts; therefore, no cause-effect relationship could be concluded. Second, there was a lack of documentation in some patient cases such as: no weight-documentation that translates into a difficulty in the estimation of the creatinine clearance of the patients and the weight-based dosing of vancomycin and teicoplanin. Third, the study took place at our medical center only, restricting the sample size of the patients reviewed and limiting the extrapolation of the results. Finally, due to the retrospective nature of the study, data on clinical improvement/harm could not be comprehensively collected and evaluated.

To improve the management of FN at our institution, it is essential to develop an appropriate protocol based on the assessment criteria utilized in the study to create a clinical pathway that will guide the prescribers. This will reduce the consumption of broad-spectrum antibiotics leading to the minimization of the emergence of resistance and an improvement in patient outocmes. Additionally, it is important to implement a dose-adjustment for antimicrobials based on the kidney function of the patients. It is also essential to use the weight-based dosing recommendation for antimicrobials requiring so (such as vancomycin and teicoplanin). Also, an adjustment of the doses of antimicrobials based on therapeutic drug monitoring (for vancomycin and teicoplanin) can be improved. It is important to procure the teicoplanin monitoring kits at the institution and to rely on the ratio of the area under the curve to the mimimum inhibiotory concentration (AUC/MIC) to of vancomycin to perform this task.

## 5. Conclusion

In conclusion, this study evaluated the appropriate management of FN at our medical center including the appropriate choice, dose, and duration of the antimicrobials used. The overall treatment of FN was mostly inappropriate compared to the assessment criteria. The choice, dose and duration of the treatment needed improvement for optimization of therapy. The choice of the antimicrobials in particular was inappropriate, with patients receiving a broader spectrum of antibiotics postcultures or an unnecessary addition of empirical MRSA coverage. The implementation of a clinical pathway for the management of FN at the institution would result in a more appropriate treatment regimen and improved patient outcomes.

## Figures and Tables

**Table 1:** Demographic and clinical characteristics of the patients *N* (% or range).

**Sex**	
Male	159 (56.8)
Female	121 (43.2)
Median age in years (range)	66 (19–96)
Median weight in kg (range)	70 (41–126)
**ANC upon presentation**	
More than 500 per μL	100 (35.7)
Between 100 and 500 per μL	107 (38.2)
Less than 100 per μL	73 (26.1)
**Classification of infection**	
Unexplained fever	137/280 (48.9)
Microbiologically defined infection	126/280 (45.0)
URTI and LRTI^a^	51/126 (40.5)
*Escherichia coli*	20/51 (39.2)
*Pseudomonas aeruginosa*	14/51 (27.5)
*Klebsiella pneumoniae*	8/51 (15.7)
Others	9/51 (17.6)
Intra-abdominal	32/126 (35.4)
*Escherichia coli*	19/32 (59.4)
*Klebsiella pneumoniae*	9/32 (28.2)
*Enterobacter faecalis*	2/32 (6.2)
*Enterobacter cloacae*	2/32 (6.2)
SSTI^b^	14/126 (11.11)
*Staphylococcus aureus*	5/14 (35.7)
*Escherichia coli*	5/14 (35.7)
*Staphylococcus epidermidis*	4/14 (28.6)
UTI^c^	11/126 (8.7)
*Escherichia coli*	9/11 (81.8)
*Klebsiella pneumoniae*	2/11 (18.2)
Others	18/126 (14.3)
Clinically defined infection	17/280 (6.1)
URTI and LRTI	11/17 (64.7)
SSTI	4/17 (23.5)
UTI	2/17 (11.8)
Bacteremia	52/159 positive cultures (32.7)
*Escherichia coli*	21/52 (40.4)
Intrabdominal infection	10/21 (47.6)
URTI and LRTI	6/21 (28.6)
UTI	5/21 (23.8)
*Pseudomonas aeruginosa*	9/52 (17.3)
URTI and LRTI	7/9 (77.8)
Others	2/9 (22.2)
*Klebsiella pneumoniae*	8/52 (15.4)
Intrabdominal infection	5/8 (62.5)
URTI and LRTI	3/8 (37.5)
*Staphylococcus aureus*	4/52 (7.7)
SSTI	2/4 (50.0)
Catheter-related infection	2/4 (50.0)
Others	10/52 (19.2)
**Type of cancer**	
Hematologic	193 (68.9)
Solid tumor	87 (31.1)
Previous hospitalization in the past 90 days	126 (45.0)
Creatinine clearance < 60 mL/min	62 (22.1)
Patients who received G-CSF^d^	148 (52.8)

^a^Upper respiratory tract infections and lower respiratory tract infections.

^b^Skin and soft tissues infections.

^c^Urinary tract infections.

^d^Growth-colony stimulating factor.

**Table 2:** Appropriateness of the treatment *N* (%).

Appropriate overall treatment regimen	90/280 (32.1)
Appropriate treatment choice	142/280 (50.7)
Appropriate treatment dose	178/280 (63.5)
Appropriate treatment duration	226/280 (80.7)

**Table 3:** Inappropriate dosing (102/280).

Teicoplanin inappropriate dosing	49/90 (54.4)
Meropenem inappropriate dosing	21/229 (9.2)
Vancomycin inappropriate dosing	6/62 (9.7)
Other antimicrobials requiring renal dose adjustment	26/102 (25.5)

**Table 4:** Anti-MRSA^a^ agent's appropriateness.

**Teicoplanin (90/280)**	
Choice	73/90 (81.1)
Dose	41/90 (45.6)
Duration	77/90 (85.5)
**Vancomycin (62/280)**	
Choice	53/62 (85.6)
Dose	56/62 (90.3)
Duration	54/62 (87.1)

^a^Methicillin-resistant *Staphylococcus aureus*.

**Table 5:** Appropriateness of treatment by site of infection and causative microorganism.

Site of infection	Appropriateness of therapy (%)
URTI and LRTI	58.8
*Escherichia coli*	55.0
*Pseudomonas aeruginosa*	64.3
*Klebsiella pneumoniae*	62.5
Others	55.5
Intra-abdominal	53.1
*Escherichia coli*	57.9
*Klebsiella pneumoniae*	44.4
*Enterobacter faecalis*	50.0
*Enterobacter cloacae*	50.0
SSTI	64.2
*Staphylococcus aureus*	80.0
*Escherichia coli*	60.0
*Staphylococcus epidermidis*	50.0
UTI	45.5
*Escherichia coli*	44.4
*Klebsiella pneumoniae*	50.0

## Data Availability

The data that support the findings of this study are available from the corresponding author upon reasonable request.

## Supporting Information

Appendix: criteria for the evaluation of the appropriate antimicrobial(s), dose, and duration of therapy.
