# Supplementary material for: Evaluation of the Management of Febrile Neutropenia in a Tertiary Care Center
Source: Can J Infect Dis Med Microbiol. 2025 Apr 8;2025:3681955. doi: 10.1155/cjid/3681955 (PMC11999742; doi:10.1155/cjid/3681955)
Supplement: Supporting Information — Additional supporting information can be found online in the Supporting Information section. [file 3681955.f1.docx]

# **Appendix:**

## Initial Management of FN:

1. *First-Line Treatment for FN 🡪* **Start** Cefepime or Piperacillin-Tazobactam or Imipenem or Meropenem
2. In case of severe beta-lactam allergy, consider initiation Vancomycin + Aztreonam
3. *If the patient presents with any of these criteria 🡪* **Add** Vancomycin or Teicoplanin:
   - *History of MRSA infection*
   - *Suspected catheter infection*
   - *Suspected SSTI*
   - *Hospitalized during the past 30 days*
   - *Received a course of IV antibiotic during the past 90 days*
   - *Septic shock (hypotension)*
4. *If the patient presents with any of these criteria 🡪* **Add** Linezolid:
   - *Confirmed resistant Gram + infection*
   - *Glycopeptide intolerance*
5. *If* *the patient presents with any of these criteria* 🡪 **Add** Amikacin or Gentamicin or Tobramycin or Ciprofloxacin or Levofloxacin:
   - *Hospitalized during the past 90 days*
   - *Received a course of IV antibiotic during the past 90 days*
6. *If patient presents with any of these criteria🡪* **Add** Colistin:
   - *History of prior colonization with XDRO*
   - *Recent ICU admission within the past 60 days*
   - *Comes from a country with known Carbapenem resistance*
7. *If the patient presents with necrotizing ulcer/ vesicular lesions* 🡪 **Add** Acyclovir
8. *If the patient presents with thrush* 🡪 **Add** fluconazole
9. *If the patient presents with esophageal findings* 🡪 **Add** fluconazole
10. *If the patient presents with severe diarrhea/confirmed C. Diff infection* 🡪 **Add** Oral Vancomycin (metronidazole PO/IV is an alternative in non-severe first episode)
11. *If the patient presents confirmed fulminant C. Diff infection* 🡪 **Add oral vancomycin +** IV Metronidazole

## Dosing Table:

| Drug | Conventional Dosing | Dosing in Renal Adjustment |
| --- | --- | --- |
| Antibiotics | | |
| Amikacin | 15-20 mg/Kg IV | 40<CrCl < 60🡪 every 36 hours  20 <CrCl< 40 🡪 every 48 hours |
| Cefepime | 2g IV every 8 hours | 30<CrCl<60 🡪 2g every 12 hours  11<CrCl<29 🡪 1g every 12 hours or 2g every 24 hours  11<CrCl 🡪 1g every 24 hours |
| Ciprofloxacin | 400 mg IV every 8 hours | CrCl< 30 🡪 400 mg every 12 to 24 hours |
| Colistin | ***Loading dose***: 9 MIU IV  ***Maintenance dose:*** 4.5 MIU IV every 12 h. or 3 MIU IV every 8 h | Dose adjustment based on total daily dose |
| Gentamycin (dose adjusted based on the concentration) | 5-7 mg/kg every day | 40<CrCl<60 🡪 every 36 hours  20<CrCl<40 🡪 every 48 hours  CrCl < 20 🡪 once |
| Imipenem | 1g IV every 8 hours  Or 500 mg every 6 hours | 30< CrCl< 60🡪 500 mg every 8 hours  15< CrCl<30 🡪 250 mg every 8 hours or 500 mg every 12 hours |
| Linezolid | 600 mg PO or IV every 12 hours | No dose adjustment required |
| Meropenem | 1-2 g IV every 8 hours | 25<CrCl<50 🡪 1-2 g every 12 hours  10<CrCl<25 🡪 500-1g every 12 hours  CrCl< 10🡪 500 -1 every 24 hours |
| Metronidazole | 500 mg IV every 8 hours | No adjustment required |
| Piperacillin-Tazobactam | 4.5 g IV every 6 hours | 20<CrCl<40 🡪4.5 g every 8 hours or 3.375g every 6 hours  CrCl<20 🡪 4.5g every 12 hours or 2.25g every 6 hours |
| Tobramycin | 5-7 mg/kg every day | 40<CrCl<59🡪 every 36 hours  20<CrCl<39 🡪 every 48 hours  CrCl <20 🡪 once |
| Vancomycin *(will be assessed based on the trough concentration)* | 15-20 mg/Kg IV every 12 hours | CrCl<50 🡪 10-15 mg/Kg |
| Vancomycin oral | 125 mg every 6 hours or 500 mg every 6 hours | No adjustment required |
| Anti-fungal | | |
| Liposomal Amphotericin B (Ambisome) | 3-5 mg/kg every day | No adjustment required |
| Anidulafungin | 200 mg on day 1, then 100 mg every day | No adjustment required |
| Caspofungin | 70 mg on day 1, then 50 mg every day | No adjustment required |
| Fluconazole | 400-800 mg IV/PO daily | CrCl <50 🡪 200-400 mg IV PO daily |
| Micafungin | 100 mg every day | No adjustment required |
| Voriconazole | 6 mg/kg IV every 12 hours for two doses, then 4 mg/kg every 12 hours | No dose adjustment required  IV is contraindicated if CrCl<50 (can use po) |
| Anti-viral | | |
| Acyclovir | 5 mg/kg IV every 8 hours | 25< CrCl<50 🡪 5 mg/kg every 12 hours  10<CrCl<25 🡪 5 mg/kg every 24 hours  CrCl<10🡪 2.5 mg |

## Duration

- 1. *If patient is afebrile and we have a focus of the infection*  🡪 **De-escalate and discontinue** the antibiotic based on each type of the infection as following:
  - Skin and Soft Tissue infection 🡪 5 to 14 days
  - Gram – blood stream infection 🡪 10 to 14 days
  - Gram + blood stream infection 🡪 7 to 14 days
  - Bacterial pneumonia 🡪 5 to 14 days
  - Candida 🡪 2 weeks after first negative culture
  - Aspergillus 🡪 minimum of 12 weeks
  1. *If the patient is afebrile and still neutropenic with no focus of infection* 🡪 **Continue** antimicrobial prophylaxis until the patient's ANC recovers over 500 and is expected to continue increasing.
  2. *If the patient is afebrile with an ANC more than 500* 🡪 **Discontinue** the Abx as long as the ANC is expected to increase.
  3. *If the patient is still febrile regardless of the ANC count* 🡪 **Continue** the Abx until the fever resolves and then re-assess the ANC count.
  4. *If* the patient is *still febrile after 72-96 hours and was not on anti-fungal prophylaxis* 🡪 **Add** fluconazole
  5. *If* the patient is *still febrile after 72-96 hours and was on anti-fungal prophylaxis refer to the following scenarios:*
  - Galactomannan (-) and CT (-) and Mucositis (-) 🡪 **Shift** to Voriconazole or Ambisome
  - Galactomannan (-) and CT (-) and Mucositis (+) 🡪 **Shift** to Ambisome or Echinocandin
  - Galactomannan (+) and CT (-) and Mucositis (+) 🡪**Shift** to Voriconazole or Ambisome
  - Galactomannan (-) and CT (+) and Mucositis (-) 🡪 **Shift** to Ambisome

## G-CSF Considerations:

- 1. *If* the patient *is receiving or has received G-CSF prophylaxis daily with Filgrastim or tbo-Filgrastim* 🡪 **Continue** G-CSF
  2. *If* the patient *is receiving or has received G-CSF prophylaxis long-lasting peg-filgrastim or eflapegrastim-xnst* 🡪 **Do not give** G-CSF
  3. *If* the patient *did not receive G-CSF prophylaxis and there are no risk factors for an infection associated complication*🡪 **Do not give** G-CSF
  4. *If* the patient *did not receive G-CSF prophylaxis and there are risk factors for an infection associated complication*🡪 **Consider** G-CSF

To note that the Risk Factors pertinent to the G-CSF criteria include the follow:

- - Age > 65 years old
  - ANC < 100
  - Anticipated prolonged neutropenia of more than 10 days (specially in patients with HSCT)
  - Documented infection
  - Fungal infection
  - Hospitalization at the time of fever
  - Previous episodes of Febrile Neutropenia
